# Paving a Shorter Path Towards Diagnosis: A Case on Adult Onset Still’s Disease from Pakistan

**DOI:** 10.7759/cureus.3255

**Published:** 2018-09-04

**Authors:** Khalid Mahmood, Samar Mahmood, Shayan Marsia

**Affiliations:** 1 Department of Medicine, Dow Medical College, Dow University of Health Sciences (DUHS), Karachi, PAK; 2 Department of Internal Medicine, Dow University of Health Sciences (DUHS), Karachi, PAK

**Keywords:** adult onset still's disease, aosd, yamaguchi criteria, autoimmune disease, serum ferritin, fever of unknown origin, erythematous rash, arthralgia, anakinra, deltacortril

## Abstract

Adult onset Still’s disease (AOSD) is a rare clinical entity with unknown etiology, characterized by arthritis, fever, erythematous rash, and other systemic presentations. We report a case of a 21-year-old male who presented with high spiking fever, dry cough, generalized body ache, arthralgia, and an erythematous rash. He was eventually diagnosed to have AOSD based on the Yamaguchi criteria, after a month of visiting three different healthcare facilities and receiving two misdiagnoses and treatment regimes not specific to his diagnosis. The patient immediately responded to prednisoloneand was healthy upon discharge.

## Introduction

Adult onset Still’s disease (AOSD) is a systemic inflammatory disorder of uncertain etiology, characterized by a presenting clinical triad of daily spiking high fevers, rash and arthritis, accompanied by biological findings of hyperferritinemia, leucocytosis with neutrophilia, and abnormal liver function tests [1]. It is considered to be a multigenic syndrome that works by way of development of a spontaneous, autoinflammatory reaction upon exposure to an environmental trigger, in the setting of genetic susceptibility. The disease mainly affects young adults and has a bimodal age distribution at 15-25 and 36-46 years of age [2]. The prevalence of AOSD is estimated to be one per 100,000 people and its rarity often makes the level of suspicion for it low among physicians. The absence of a specific diagnostic test for AOSD makes its diagnosis one of exclusion and the preceding differentials numerous, with the reported time from the onset of symptoms to diagnosis known to vary from a mean of four months, to even up to three years in one case [3]. A number of infectious, autoimmune, neoplastic, and drug-related hypersensitivity reactions can mimic the presentation of AOSD, necessitating the development of several sets of different classification criteria for it over the years. The most widely used one is by Yamaguchi et al. developed in 1992 [4-5].

We report a case of AOSD in a 21-year-old Pakistani male belonging to the lower socioeconomic class, whose journey to correct diagnosis and treatment took him through three healthcare facilities over an arduous month, comprising two misdiagnoses and treatment regimes not specific to his diagnosis. In a country where 24.3% of the population lives below the poverty line [6], where an overwhelming majority cannot afford the specialized healthcare that they need [7], and where the approximate incidence for AOSD has been quoted at just 1.3 cases per year [8], raising awareness of the disease and its diagnostic challenges will direct physicians towards its correct early diagnosis and streamlined intervention—ensuring quick, efficient treatment, and thereby prevention of complications.

## Case presentation

A 21-year-old Pakistani male presented with unresolved, high spiking fever for four weeks, associated with episodes of moderate headache, mild, dry cough, generalized body ache, arthralgia and later, an erythematous rash over his back and limbs. The fever reportedly developed a month before the current hospital visit while the patient was on vacation in his hometown, in the rural area of Jacobabad. It began as episodes of high grade fever and progressed to a more continuous pattern with chills accompanying both phases. There were multiple bouts of fever through the days and nights which would only partially and temporarily respond to acetaminophen or ibuprofen. There was no previous history of similar fever break outs. The arthralgia was mostly confined to the knee and ankle joints. Upon first presentation to a healthcare facility in the nearby small city of Sukkur, the patient was hospitalized for fever and treated with intravenous antibiotics (ceftriaxone and meropenem) along with paracetamol for pain and artemether for suspected malaria. The various lab investigations conducted, including those for the malarial parasite, hepatitis B surface antigen, hepatitis C antibody and HIV 1 and 2 antigens, all came out normal, as did his X-ray chest, echocardiography, and ultrasound of the abdomen. After a two-week stay and unsettled fever, the patient was referred to a healthcare center in Karachi with tertiary healthcare facilities where he was treated with further antibiotics as a potential case of enteric fever, but to no relief.

During this third presentation, the examination revealed a weak looking, fatigued male to us with a fever of 39.0°C. The erythematous rash over his back and limbs, noted first upon this third presentation, had possibly gone unnoticed before due to his dark colored skin. There were no other remarkable findings from the other systems, including no sore throat or synovitis. Hematological investigations showed elevated neutrophils (82%), a total leukocyte count touching the upper limit of normal, disturbed liver function tests with elevated alanine transaminase (124 U/L), and a remarkably high level of C-reactive proteins (13.5 mg/dl). Renal and coagulation profiles were normal, as were blood and urine cultures and the chest X-ray. The anti-cyclic citrullinated peptide, antinuclear antibody (ANA), and rheumatoid factor (RF) were all negative as well. As all these systemic investigations continued to come out normal, the diagnosis of exclusion kept increasing the chances of this being a case of AOSD. Based on this suspicion, the serum ferritin levels were carried out and found to be markedly elevated (2698.00 ng/mL). Additionally, the ultrasound of the abdomen revealed benign looking, small lymph nodes (para-aortic and mesenteric) that were not noticeable on the previous scan, as well as mild hepatosplenomegaly.

Based on his clinical features and review of the laboratory evaluations, the differentials considered this time were: a resistant infection like that by cytomegalovirus (rooted out by the lack of a sore throat and no monocytosis in the laboratory reports), thyroiditis (excluded due to the absence of an enlarged and tender thyroid), lymphoma, and Still’s disease. Although serum ferritin is raised in both lymphoma and AOSD, a diagnosis of lymphoma usually follows findings of more significant lymphadenopathy and hepatosplenomegaly, variable weight loss and raised lactate dehydrogenase (LDH) levels on investigations, along with other signs and symptoms. The absence of the aforementioned, coupled with the patient’s immediate response to the first dose of steroids with the settling of fever and improved wellbeing, all pointed towards AOSD and away from lymphoma—further confirmed by the Yamaguchi criteria [5].

The patient was treated with oral prednisolone in tapering doses, starting with 60 mg per day. As mentioned, he responded to the very first dose with the settling of fever. He went home and returned after two weeks, in an absolutely normal state and did not have a recurrent bout of fever. He was advised to come back in case of any relapse.

## Discussion

The global incidence of AOSD is approximately 0.6 per 100,000 people per year [9] with an interestingly higher incidence obtained from Canada and from a series of fever of unknown origin (FUO) patients from Turkey [8]. Most studies have described either a female preponderance or equal distribution between the sexes [10] with a male preponderance reported only from the South Asian population [11]. This disparity may very well be due to the fact that significantly lesser females seek medical care in the Third World countries in the region, thus counting for unreported cases and misrepresented statistics. The pathogenesis of the disease remains unclear, but its correlation with several cytokines like tumor necrosis factor-alpha (TNF-α), interleukin (IL)-6, and IL-18 has been established, along with the collective role of genetic, infectious, and environmental factors in its course—leading to its reclassification as a polygenic autoinflammatory disorder in the past decade [12].

Three broad patterns of AOSD have been described: monocyclic with complete remission, which was seen in our case; polycyclic with recurrence of systemic and articular flares, separated by periods of remission; and a chronic pattern, most prone to joint destruction [12]. Alternately, the disease is classified into two subtypes—the rheumatoid arthritis (RA) subtype with severe articular involvement and the non-RA subtype, characterized by macrophage activation and systemic manifestations. Our case belonged to the non-RA subtype which is generally more common, as backed by its overwhelming 77.46% prevalence amongst the AOSD patients in Ichida et al.’s study as well [13].

The diagnosis is of exclusion and is confirmed by the most extensively used Yamaguchi criteria (1992). This includes five or more criteria; two or more of which can be from the list of major criteria (arthralgia for more than two weeks, fever more than 39°C for more than one week, typical rash and leucocytosis for more than 10,000/mm3 including more than 80% granulocytes), with the rest as minor ones (sore throat, lymphadenopathy or splenomegaly, liver dysfunction, negative RF, and ANA). The three conditions to be specifically excluded prior to diagnosis are infections, malignancies, and rheumatic diseases [4]. From the above—fever (seen in 60%-100% patients, highest in the evenings), sore throat, and arthralgia (in 70%–100% of patients; mainly in wrists, knees, and ankles) are the most typical clinical features. Interestingly, sore throat was the only symptom that our patient did not present with, while he fulfilled all the other seven criteria. This portrays how straightforward the workup would have been, had the prior physicians been aware of the prevalence of the rare condition of AOSD and the use of the above stated criteria.

Other associated findings have been seen to have a varying prevalence amongst patients of AOSD from different countries. Ishaq et al.’s review showed how weight loss, for example, was seen in 84.6% of AOSD patients in Thailand, but only in 30.8% of Pakistani patients whereas pericarditis was seen in patients from France and USA, but none of those from Pakistan [11]. This again points towards an aspect of AOSD that needs to be further worked upon—to determine whether these differences are significant and the underlying causes for them.

Moreover, the most promising laboratory investigation to date—the glycosylated ferritin—has been useful but not definitive, as it is also a common feature of malignancy and infection and only 43.2% sensitive. The disproportionate increase in ferritin levels is thought to be due to cytokine secretion induced by the reticuloendothelial system or hepatic damage, although most cases present with increased levels, without any evident liver damage [12]. As research on AOSD continues, other novel biomarkers with diagnostic potential continue to emerge. Recent studies have portrayed higher levels of germinal center kinase-like kinase (GLK)-expressing T-cells in sera of AOSD patients, of calcium binding protein S100A8/A9, IL-18 and mean calprotectin levels—all seen to decrease after treatment [9]. However, these require evaluation in larger studies before any conclusive decision can be made regarding their diagnostic potential.

Furthermore, as the path towards reaching the AOSD diagnosis is so lengthy, it is natural that several prior diagnoses are made along the way, in as many as 80% of the patients [14]. Our patient was misdiagnosed as a case of malaria and enteric fever, the latter being the perceived diagnosis for 6.66% of AOSD patients in a previous Pakistani study as well [10]. Other cases in the past have been treated as rheumatic fever, infectous mononeucleosis, systemic lupus erythmatosis, polyarthritis nodosa, Henoch-Schönlein purpura, tuberculosis in most Third World countries where it is prevalent, Lyme disease and gonococcal meningitis—all reportedly due to the overlapping symptoms of fever, sore throat, and arthralgia [15]. This further highlights the need for increasing the awareness of AOSD amongst physicians so it strikes their minds during evaluation of a case that points in the direction and saves them, the healthcare facilities and the patients, the added financial burden, and misery of unnecessary treatment.

Regarding the treatment, mild cases can be treated with nonsteroidal anti-inflammatory drugs (NSAIDs), but more than 80% of AOSD patients do not achieve remission and require the use of corticosteroids. Diseases modifying anti-rheumatic drugs (DMARDs) like methotrexate are sometimes used to control acute symptoms and biological agents such as anti-TNF-α and IL-1 antagonists are considered for those not responding to the above [9]. Patients with systemic AOSD are more likely to be responders to first-line corticosteroid therapy. In the case of refractory systemic AOSD, IL-1 antagonists (mostly anakinra) should be considered first as they have proved to be dramatically more efficient for systemic symptoms than for articular features. AOSD during pregnancy has most successfully been treated with anakinra with the children born at term and healthy as well.

In addition, amongst the complications of AOSD the most life-threatening ones are: reactive hemophagocytic lymphohistiocytosis, disseminated intravascular coagulopathy, myocarditis (early complication occurring within the first year after the disease onset and affecting younger patients, mostly males), thrombotic thrombocytopenic purpura (marked by acute visual loss), and diffuse alveolar hemorrhage [16]. Although the diagnosis of AOSD necessitates the exclusion of neoplasms, there have been case reports in more recent times suggesting the presence of AOSD or an AOSD-like syndrome in the course of various malignant diseases including leukemia, lymphoma, breast, thyroid, and esophageal cancers. This has raised questions on whether these are coincidental processes or if AOSD is a paraneoplastic condition, opening yet another avenue for more work to be done regarding the disease [17].

Finally, regarding the limitations—specific tests for the exclusion of cytomegalovirus infection via antibodies and lymphoma via immunological markers could not be carried out, as would have been ideal. As mentioned, the patient belonged to the lower socioeconomic group and reported to a healthcare facility in a similar setting, after having gone through extensive laboratory investigations already—so the prescribed investigations had to be kept to a minimum, with the differentials having to be excluded on the basis of the performed tests and the presence or absence of clinical signs and symptoms. 

## Conclusions

Thus, we see the rare condition of AOSD and its clinical presentations, subtypes, laboratory findings, classification criteria, most frequent differentials, treatment, and complications. This article highlights the need for future work to be done regarding the various global incidences of the numerous associated symptoms of AOSD, the several new biological markers with diagnostic potential coming to the forefront, on its proposed association with various malignancies and most importantly, the promotion of awareness of the prevalence of the condition amongst physicians, so that the path towards its diagnosis can be shortened and the numerous misdiagnoses can be avoided.
